# Unreported and unaddressed: Students with disabilities experience of school violence in Zambia

**DOI:** 10.4102/ajod.v11i0.849

**Published:** 2022-03-30

**Authors:** Janet Njelesani, Jessica Si, Drake Swarm

**Affiliations:** 1 Department of Occupational Therapy, New York University, New York, United States of America

**Keywords:** qualitative, Zambia, inclusive education, bullying, violence, disability

## Abstract

**Background:**

Violence against school children is a prevalent global issue. Despite the high prevalence of school violence in Zambia, there is limited research on students with disabilities’ experiences of school violence.

**Objectives:**

Guided by the socio-ecological model for bullying, the aim of this study was to understand students with disabilities’ experiences of school violence in the Lusaka and Southern provinces of Zambia.

**Methods:**

A qualitative descriptive study was conducted with 14 purposively sampled boys (*n* = 6) and girls (*n* = 8) with disabilities. Data were generated using semi-structured interviews and child-friendly methods. Child-friendly methods were co-constructed with Zambian youth with disabilities in order to ensure cultural appropriateness and included vignettes, cartoon captioning, photograph elicitation, drawings, and sentence starters. Qualitative data were analysed by thematic analysis.

**Results:**

The themes illuminated that violence against students with disabilities occurs frequently but goes unaddressed. Moreover, students with disabilities were being blamed for causing the violence, and therefore, considered a risk to others. Participants reported that they turn to trusted teachers for support.

**Conclusion:**

This study illuminates the violence students with disabilities experience within the Zambian education system, with implications for school policies and programmes, peer education, and teacher training to create a safer education environment for students with disabilities.

## Introduction

Violence against school children is a prevalent worldwide issue (Hui et al. 2018; Njelesani 2019; Njelesani et al. 2018), with children with disabilities being 3.7 times more likely to experience violence than their non-disabled peers (Fleming & Jacobsen 2010). In this study, ‘school violence’ is defined as ‘all forms of physical, or mental violence, injury and abuse, neglect or negligent treatment, maltreatment or exploitation including sexual abuse’ (Office of the United Nations Commissioner for Human Rights 1989). School violence leads to many negative consequences including increased risk of depression, suicide attempts and low educational attainment (Centers for Disease Control and Prevention 2014). The increased violence against students with disabilities is directly related to their exclusion in schools and the community (Mepham 2010). Therefore, the right to feel safe in schools is dependent upon inclusion. However, research into inclusive education has largely not considered child protection issues. Therefore, greater research is needed to understand how to prevent and address school violence against students with disabilities. Research studies on the effects of school violence and the possible methods of mitigating this global issue for students with and without disabilities have primarily been conducted in high-income countries, with less data generated from low and middle-income countries (LMICs). As a result of the limited research on the experiences of school violence among students with disabilities from LMICs, this qualitative descriptive study explores the experiences of school violence among students with disabilities attending mainstream schools in the Lusaka and Southern provinces of Zambia.

### Disability in Zambia

There are many educational policies in Zambia, for example, *The Education Reform Document* (1977), *Focus on Learning* (1992) and *Educating Our Future* (1996) that lay the foundation for current practices in inclusive education (Chitiyo & Muwana 2018). These policies are supported in the *Disability Act of 2012*, promoting access to education (Republic of Zambia 2012). Historically, students with disabilities attended segregated special schools in Zambia; however, there has been a move to inclusive education in mainstream schools over the past few decades (Chitiyo & Muwana 2018). However, despite the existing legislation and increasing awareness and efforts to include all learners, educational opportunities for children with disabilities within the Zambian education system are more limited than for their non-disabled peers (Njelesani et al. 2014). Fewer children with disabilities (80.8%) than non-disabled children (89.9%) reported having attended school (UNICEF 2016). This education gap leads to inequities for students with disabilities with harmful long-term effects. The lack of inclusion is because of many factors, including poverty (Peele, Gill & Wainscott 2020), inconsistent implementation of laws and policies at the local level, and disability stigma stemming from culturally driven fears and misunderstandings that stereotype children with disabilities (UNICEF 2018), ultimately denying them their rights and resources.

### Violence against children in Zambia

Despite Zambia’s adoption of legislation that bans corporal punishment in schools, a high prevalence of violence is reported. In 2014, Zambia conducted the Violence against Children Survey (VACS), a cross-sectional household survey that produced a national-level estimate of the physical, sexual and emotional violence experienced by the youth in Zambia. The results found that approximately half of boys and girls experienced one or more forms of sexual, physical and emotional violence in their childhoods, with physical violence being the most common. The first incidents of violence occurred between ages 6–11 for girls and ages 12–17 for boys (VACS 2014). No data were disaggregated according to disability or impairment type; however, given the cultural stigmas against disabilities it is likely that violence rates will be higher against boys and girls with disabilities.

### Violence against students with disabilities

School violence is widespread and institutionalised in Zambia, where corporal punishment is considered as an inevitable part of school life. Despite legislation aiming to prevent corporal punishment, there is reluctance from education authorities to address the problem and prosecute perpetrators (VACS 2014). This previous research provides a broad picture of the problem of school violence in Zambia. Students with disabilities face even greater victimisation (Fleming & Jacobsen 2010); however, there is a dearth of research on school violence against students with disabilities in Zambia. Furthermore, research globally has failed to report feelings of students with disabilities and perspectives on their experiences. Children with disabilities have the right to participate in research, so their priorities, needs and experiences are included. This right is upheld by Article 12 of the United Nations Convention on the Rights of the Child (CRC), which indicates that every child should have the right to express their views freely (CRC 1989).

### Research aim

The aim of this study was to understand the experiences of school violence among students with disabilities in the Lusaka and Southern provinces of Zambia. Including students’ perspectives provided youth with disabilities opportunities to talk about their past and current school violence experiences. The research findings allow for a greater understanding of the context in which students with disabilities experience school violence in Zambia, which is essential for the development of intervention methods. These intervention methods established with the consideration of students’ personal, social, educational and cultural context are more effective (Silva et al. 2017).

### Theoretical framework

The socio-ecological model for bullying was used as the theoretical framework to better understand the violence against students with disabilities, from the individual (e.g. age, gender, and type of impairment), relationship (e.g., peers, parents, teachers), community (e.g. school policies), and societal levels (e.g. cultural norms). The model recognises that school violence does not occur in isolation between perpetrators and victims as individuals are affected by their surroundings. Therefore, interventions also need to target broader social environments (Espelage & Swearer 2009). Previous studies using the model have shown that peer attitudes and a negative school climate are strongly associated with school violence (Swearer et al. 2006). An ecological approach to examine the school system in which students with disabilities attend in Zambia can provide a more holistic understanding of what is needed for successful interventions.

### Reflexivity

The authors have considerable experience in child protection qualitative research and experience working in Zambia, including working with the local youth with disabilities and Zambian research assistants in this study. As the authors of this article are located outside of Zambia, and their outsider perspectives and experiences influenced the research, they worked in collaboration with Zambian researchers and an advisory board of Zambian youth with disabilities to mitigate concerns, including power dynamics. Data were collected by the first author and a team of Zambian researchers. The first author organised the research collaboration and has sustained it over 10 years (Njelesani et al. 2013).

## Methodology

### Study design

This study utilised a qualitative descriptive approach. It is an appropriate study design to explore people’s experiences and obtain descriptions of phenomena (Sandelowski 2000).

### Recruitment

Students with disabilities attending primary and secondary mainstream schools where inclusive education was offered were included in this study. Participants were recruited from Lusaka and Southern provinces, the two largest provinces in Zambia. Purposive sampling was utilised to recruit students of varying ages, genders and impairment types. The participants were referred to the research team by the principals of the schools. Participant inclusion criteria included being enrolled in a mainstream school and self-identify as having a disability. The recruitment was conducted until data saturation occurred (i.e., new information was not reported).

### Ethical considerations

The study received ethics approval from the Institutional Review Boards at New York University and the University of Zambia (reference number: 2018-Jul-001a20). We obtained participants’ verbal consent before data collection. In order to protect the children during data collection, two people were present, including at least one Zambian researcher of the same gender as the child. The locations for data collection were selected, taking into consideration participants’ accessibility needs, safety, convenience, and anonymity. The reports of abuse were notified to local authorities by the Zambian research assistant following consent from the participant and their parent. The participants’ confidentiality was held to the highest importance, and we used pseudonyms in documenting the data and throughout this study. All participants were provided monetary compensation for participating.

### Data collection

Data were collected in schools, at afterschool programmes and at disability organisations. The participants participated in two 60-min data collection sessions, where data were generated through child-friendly methods and semi-structured interviews. Child-friendly methods were included to engage children and encourage expression given the power differences and stigmatising topics (Teachman & Gibson 2013). Child-friendly methods were co-constructed with Zambian youth with disabilities in order to ensure cultural appropriateness and included vignettes, cartoon captioning, photograph elicitation, drawings and sentence starters. Data collection methods were first piloted with three students and modified to be more age and culturally appropriate. Two data collection sessions were conducted with each participant 3–5 days apart. Two sessions were conducted to build rapport with the participants as data collection addressed sensitive and stigmatising information.

### Data analysis

Using thematic analysis (Braun & Clarke 2019) and the qualitative data management software Taguette, data generated from interviews and child-friendly methods were analysed by all authors. Data were first uploaded into Taguette. Data from the visual child-friendly methods (i.e., drawings) were treated as visual text, and a written description of each picture drawn as provided by the participant was uploaded (Prosser 2007). Each author familiarised themselves with the data by reading the interview transcriptions line-by-line multiples times and writing a memo that noted ideas and insights of what they were learning from the data. Then, the team collaboratively generated a list of a priori codes (e.g., school violence norms) that came from the research aim, the literature review, and the socio-ecological model for bullying. Data were coded using the a priori codes, and then inductive codes (e.g., youth seen as the risk) were developed from the data, added to the codebook and coded across the data set. During multiple team meetings, inductive codes were modified and defined. Following coding, we reviewed all of the coded data, and codes were grouped into broader potential themes. Using a thematic map, we reviewed all of the potential themes to determine whether themes were representative of the coded extracts and the entire data set. After multiple team discussions, reflections and refinements, themes were finalised, where each theme was named and defined. Trustworthiness was ensured throughout the process by memo writing, using a theoretical framework to guide analysis, thoroughly detailing data collection procedures and analysis, and presenting themes with quotes from the participants.

## Findings

### Participant characteristics

This study included 14 participants (six boys, eight girls), with age ranging from 12 to 20 years, and were enrolled in Grade 4 to Grade 12. All participants were students with disabilities enrolled in a mainstream public school in Zambia. The participants had a range of impairments, including identifying as blind, deaf and having physical impairments.

### Themes

The themes illuminate that (1) school violence is a regular occurrence, (2) school violence goes unaddressed, (3) students with disabilities are considered the risk to others, and (4) students with disabilities identify one person as their primary source of safety.

#### Violence regularly occurs

All 14 participants spoke of violence regularly occurring against them at school. The majority of the reported violence was emotional and was perpetrated by peers. This emotional violence from peers was reported to be very hurtful: ‘They say look at this cripple. When I start walking people think its comedy. Those words really hurt, they can cause someone to start thinking negative of himself’ (Ian, 12, male). Peers also perpetrated physical violence. When asked to speak about the pictures they drew during data collection, one participant reported:

‘It’s a picture of someone pushing me. He pushed me very hard for no reason. I fell down the stairs near one of the classrooms. I cried, and people laughed because the boy who pushed me was small, but I was in a lot of pain.’ (Paul, 20, male)

Another said of their drawing: ‘one of the boys has a stick, and he is using it to hit me on the football field’ (Mary, 16, female).

The participants also spoke of how teachers inflicted emotional abuse and greater corporal punishment than non-disabled students: ‘the teachers make fun of me. They say teacher W is my wife’ (Kalonde, 18, male) and ‘they are rude, and they say words which will hurt you and the words ruin your whole day’ (Ian, 12, male). Several participants spoke of the physical punishments they received as a result of their difficulty in completing school tasks because of their disability:

‘I’m a slow writer [*note taking from the blackboard*], and the teacher is faster than me. After the teacher is done, the teacher rubs [*erases the notes*] on the board. I ask, “I am not yet done,” then the teacher says, “Why are you being slow” and forces me to sit on the floor for an hour.’ (Bupe, 10, female)

One 13-year-old deaf female student disclosed the sexual violence she experienced from a staff member, which led to pregnancy. When prompted on the actions taken by the school, the girl indicated that the school authorities blamed her, and her behaviour was highlighted in a school assembly focusing on the consequences of bad behaviour. Her experiences exemplify the following two themes of violence not being addressed and students with disabilities blamed for the assaults.

#### Violence goes unaddressed

Violence was not reported or addressed when it occurred in schools, as expressed by one student who had been victimised: ‘I don’t do anything. I don’t report them to the teacher’ (Gabrielle, 15, female). Even if a student reported an incident, there was a disproportion of action or repercussions that occurred. One of the students talked about how a teacher neglected to act: ‘I would report to the teachers on duty, but some teachers would walk away’ (Kalonde, 18, male). Another student wanted the non-disabled students to receive the same punishment as students with disabilities suffered: ‘they should take them to the police station like I was taken to the police station’ (Peter, 14, male). Not only there was a lack of response to stop school violence from occurring, but the students who reported also experienced negative consequences from teachers: ‘they chase us away, and sometimes they even laugh at us’ (Wakumbo, 14, female).

#### Students with disabilities are considered the risk

The participants reported that when incidents of school violence occurred, teachers blamed the students with disabilities, telling them it was their fault, and that they were the problem and disturbance:

‘One day when I was playing a boy hit me with a stick. When I complained to the teacher, the teacher said I could not play football anymore. He said I am the one who disturbs the team.’ (Taona, 17, male)

Other students spoke of similar experiences: ‘The teachers don’t let me play. Even when I wanted to join the chess club, they said I could not because I can’t hear’ (Halima, 15, female) and ‘I want to play. They say I will hurt them with my wheelchair and I can’t kick a ball’ (Chanda, 10, male).

Being blamed for the violence that occurred was reported in other school interactions, as one child spoke of when any incident happened, it was students with disabilities who were blamed:

‘Some of the normal kids steal, and then the teachers think it is us. That makes me very angry. So now we stay near each other during break time and we stay near our class so that nobody can blame us.’ (Paul, 20, male)

When the students with disabilities were blamed, they often experienced greater violence, as one student spoke about mistakenly being brought to local authorities:

‘The teachers said I stole Mrs. M’s food from her office and that I took a ball from the office to play with. Mrs. M took me to the police station, and the police beat me. She told the police to beat me. They beat me with a stick.’ (Wakumbo, 14, female)

#### Safety is not a place but a person

During data collection, the participants were asked to describe settings where they felt safe and most scared. One’s home was identified as a location of safety, while inaccessible sites such as latrines were considered unsafe. The inaccessibility of places and activities fostered environments of exclusion leading to potential violence. One student spoke ‘playground is not good for us, especially when there are boys. I feel scared there because they don’t want us there, and they bother us when we go there’ (Triana, 13, female). Another student spoke of avoiding going to use the latrines: ‘I don’t feel safe going outside because anything can happen when I am trying to access that area’ (Ian, 12, male).

Although the participants identified locations, more often the students identified peers or one teacher who provided them the feeling of safety: ‘I feel safe at school when I am in class or with other deaf kids’ (Victoria, 13, female), and ‘I go to Teacher Mary’s house with the other kids on weekends, and we spend time together there’ (Paul, 20, male). Other children expressed their appreciation for teachers who protected and cared for them: ‘she buys us uniforms and clothes. She is the only one who does that for us’ (Wakumbo, 14, female).

## Discussion

The findings overall illuminate that students with disabilities who participated in this study regularly experienced violence because of their physical and functional differences, and they were considered a risk to others and portrayed as the problem. This violence was predominately perpetrated by non-disabled school peers and went unaddressed by school teachers. To our knowledge, this is one of the first studies to explore the experiences of school violence against students with disabilities in Zambia. The study’s findings support the limited existing research in the field of violence against students with disabilities, where students with disabilities are often marginalised and victimised (Kabwe, Mandyata & Chakulimba 2020).

Children with disabilities are 3.7 times more likely to experience violence than non-disabled peers (UNESCO 2016). The amount of violence that students with disabilities experience is believed to be underestimated. This study findings support that notion as the themes identified was that violence occurs regularly but goes unreported or unaddressed. When students reported an incident of violence, there was a disproportion of action or repercussions made. In this study, not only there was no action taken by teachers but also some teachers discriminated against and abused students with disabilities. Participants described different forms of violence in their interviews; however, they did not always report their experiences to authorities. This was in part, because of the fear of teachers and punishments they may receive and as authorities in the community may not be accessible. For example, persons who are Deaf may not have access to report what happened because police do not use sign language (Senne 2016).

When considering the association between social-ecological factors in the school environment in Zambia, and because all of the factors influence school violence in either direct or indirect ways, interventions must involve students, non-disabled peers, teachers, parents and the community. Interventions aimed at non-disabled peers should include peer education at the individual level that impacts knowledge, attitudes and beliefs. Previous studies have found that education that leads to an increased awareness of disability is significantly correlated with positive shifts in attitudes towards disability (Parasuram 2006). A study on students’ social experiences with low vision in Lusaka and Mbala districts in Zambia indicated that implementing sensitisation and advocacy interventions helped to reduce stigmatisation, discrimination, teasing and bullying. Furthermore, the programme provided greater social inclusion for students with low vision (Kabwe et al. 2020). Without such peer education, greater stigma and discrimination may lead to further societal exclusion, bullying, aggression, feelings of shame and disengagement of students with disabilities from schools and the society (Earnshaw et al. 2018). Previous studies in Zambia have found that to avoid being victimised, students will miss classes, which leads to lower performance in school (Siziya, Rudatsikira & Muula 2012). We saw the lack of attendance in this study as recruiting participants was difficult, where many children with disabilities were not at school despite being enrolled. Within the school curriculum, teaching respect for one another, and promoting positive values and attitudes towards peers should be included (Bradshaw, Waasdorp & Johnson 2015). Thus, peer education, other whole-school interventions and the teacher training will be essential components of anti-violence programmes in the schools studied, with concurrent work to strengthen policy at the community and society levels.

Although students identified some safe locations at school, most identified a person, particularly a teacher, who helped them feel safe. For students, a strong sense of belonging is dependent on high-quality relationships with teachers, as the quality of interpersonal relationships and how teachers, students, and parents relate to one another sets the tone for the school (Mitchell, Kensler & Tschannen-Moran 2018). This finding indicates that in the schools studied more supports need to be put in place for students with disabilities as there is a shortage of qualified inclusive education personnel across Zambia. Furthermore, not all mainstream teachers provide the same level of support to their students, and not all have received the same level of training, especially regarding the rights of students with disabilities and their increased risk of violence. At the school level, teachers and staff could benefit from in-service training on disability, risk factors to violence, how to address and report incidents, and non-violent methods to address the violence that occurs in the school. Currently, Zambia’s pre-service teaching programmes include a curriculum that educates teachers on providing support for students with disabilities. These programmes have proven useful to inform teachers on how to work with all students and have included educational psychology courses to strengthen student teachers’ knowledge and counselling skills to address psycho-social challenges (Chitiyo et al. 2015). Adding school violence as a compulsory topic in this pre-service teacher training could help ensure that students have more teachers who understand their rights and needs for a safe learning environment.

Critical to understanding how broader social forces and structures influence the violence experienced by students with disabilities in this study is that Zambia has no single national policy against school bullying or violence, leading to varying policy implementation at the school level. Bullying is strictly prohibited in some private schools, warranting suspension or expulsion, while most public schools do not implement such policies (Siziya et al. 2012). The lack of clear and coordinated national school policies and guidelines was a key finding of a recent study on inclusive education in Zambia (Ngulube, Njelesani & Njelesani 2020). However, as suggested by Ngulube’s study, teachers will favour implementing policies that respect the rights of children with disabilities with appropriate administrative, material and school leadership support. Therefore, clear policy statements for school violence backed by technical, legal, and financial resources and the involvement of administrators, teachers, students and families are essential in creating safe schools.

When considering how to address the Zambian social contexts within which the violence occurs, how cultural discipline is practised in Zambia must also be regarded. Teachers may favour corporal punishment and view it as an appropriate way to discipline and protect children. While we did not interview teachers, the literature suggests that teachers may not believe that their actions were perpetrating violence as they were acting on behalf of parents at the school (Kabungo & Munsaka 2020). The key to ending corporal punishment in Zambia requires work across ecological systems, including educating teachers on prosocial methods of discipline and advocating for implementing the existing legislation that bans corporal punishment with the support of parents, school staff, and local and religious leaders (Breen, Daniels & Tomlinson 2015).

### Limitations and future research

Limitations of the study were a small participant pool. The participants needed to self-identify as having a disability, and thus there was no open recruitment as referrals only came from school principals. The lack of attendance as many students with disabilities were not in schools may have resulted in fewer opportunities to generate data. Having access to out-of-school students and alternative referral pathways for students (e.g. an opportunity to self-refer) would increase the quality and depth of data. Also, only students were interviewed and future research could involve a variety of stakeholders, including teachers, parents and school administrators, across a larger subset of Zambia’s population and not focusing solely on two provinces as micro-cultural differences can exist.

## Conclusion

While there are government policies in place that support a safe education for all in Zambia, school violence remains a prevalent issue. Students with disabilities are at greater risk of being marginalised, bullied and violated than their non-disabled peers. Study findings suggest that violence in schools goes unaddressed with little or no repercussion for perpetrators, leading to lower educational and societal participation from marginalised students. Implications from the findings highlight the need for strengthening actions across systems to address the school violence experienced. For example, as students generally identified only a single person who advocated for them, educator training should include disability inclusive content for teachers and staff. Furthermore, as research on school violence against students with disabilities in LMICs is scarce, there is a need for further research to determine how to best direct resources, implement policies and create culturally contextual specific programmes to address school violence.
